# Arachidonic acid metabolism in spinal cord injury

**DOI:** 10.1016/j.jare.2025.11.058

**Published:** 2025-11-28

**Authors:** Xue Yao, Xinjie Liu, Wenbin Cai, Guangzhi Ning, Xu Zhang, Kailiang Zhou, Shiqing Feng

**Affiliations:** a Tianjin Key Laboratory of Spine and Spinal Cord, Tianjin Institute of Orthopeadic Innovation and Translation, International Science and Technology Cooperation Base of Spinal Cord Injury, Department of Orthopedics, Tianjin Medical University General Hospital, International Chinese Musculoskeletal Research Society Collaborating Center for Spinal Cord Injury, Tianjin 300052, China; b Tianjin Key Laboratory of Metabolic Diseases, Department of Physiology and Pathophysiology, The Province and Ministry Co-sponsored Collaborative Innovation Center for Medical Epigenetics, Center for Cardiovascular Diseases, Research Center of Basic Medical Sciences, Tianjin Medical University, Tianjin 300070, China; c Department of Biophysics, School of Basic Medical Sciences, Peking University Health Science Center. State Key Laboratory of Vascular Homeostasis and Remodeling, Peking University, Beijing 100871, China; d Department of Orthopedics, The Second Affiliated Hospital and Yuying Children’s Hospital of Wenzhou Medical University, Zhejiang Provincial Key Laboratory of Orthopedics, Wenzhou 325027, China

**Keywords:** Spinal cord injury, Arachidonic acid, Cyclooxygenase, Lipoxygenase, Eicosanoids, Endocannabinoids, Isoprostanes

## Abstract

**Background:**

Spinal cord injury (SCI) initiates a cascade of secondary pathological processes significantly impairs functional recovery and leads to complications such as neuropathic pain. Arachidonic acid (AA) and its metabolites play crucial roles through various metabolic pathways. These pathways contribute to neuroinflammation, tissue damage, and functional impairment following SCI.

**Aim:**

We aim to provide a comprehensive outline on AA metabolic pathways, enzymes and signaling cascades in spinal cord injury pathophysiology, in order to develop new perspectives in treatment and diagnosis.

**Key scientific concepts:**

We present a comprehensive review on AA metabolism and pathways in the different pathology process of SCI, such as neuronal loss, demyelination, neuropathic pain and neuroinflammation with different cell types involved. We also discussed the complex interactions of AA metabolites pose challenges for the development of effective therapies. Finally, we outlooked future research directions aimed at optimizing AA-targeted treatments and expanding their clinical applicability to enhance recovery in patients with SCI.

## Highlights

• Arachidonic acid (AA) and its metabolites drive secondary injury and inflammation following spinal cord injury (SCI).
• The COX, LOX and P450 metabolic pathways of AA exacerbate neuronal loss, demylination, neuroinflammation and neuropathic pain after SCI.
• The complex interactions of AA metabolites impede developing effective therapies, requiring further research to optimize treatment strategies.

## Graphical abstract

**Figure:**
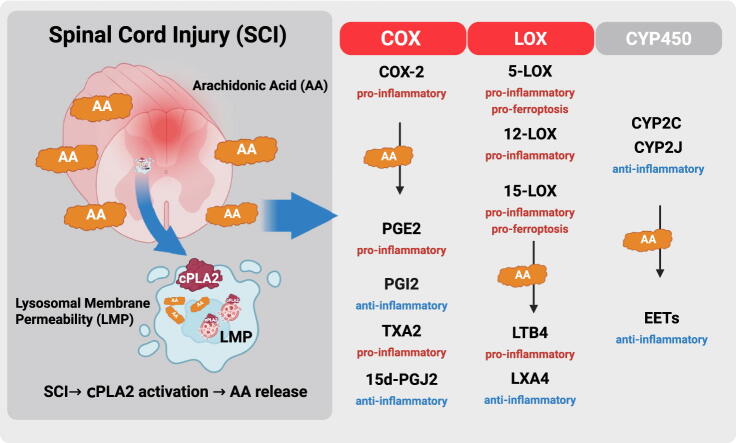

## Introduction

Current therapeutic interventions for spinal cord injury (SCI) demonstrate limited efficacy. This condition induces severe and permanent physical, neurological, and psychological impairments, substantially diminishing quality of life [1,2]. The global increase in SCI incidence imposes considerable societal and economic burdens on healthcare systems and social services [[3], [4], [5]]. SCI classification distinguishes between traumatic and non-traumatic etiologies [6], with traumatic SCI predominating. Primary mechanical trauma typically results from events like falls, motor vehicle accidents, or sports injuries, causing immediate cellular death, blood-spinal cord barrier disruption, and extracellular matrix degradation. These primary effects are compounded by secondary pathological processes—including inflammation, oxidative stress, and extensive nerve damage—that critically drive injury progression and severity, thereby hindering recovery [[7], [8], [9]].

Arachidonic acid (AA) serves as a key signaling molecule in cellular injury responses [10]. Exogeneous source of AA is from animal food and from vegetable oil linolic acid and also from lipid droplet [11]. Endogeneous AA is stored within the phospholipid bilayer, and is released during cellular stress or damage via cytosolic phospholipase A2 （cPLA2) activity [12]. Metabolism of free AA occurs primarily through four enzymatic pathways: cyclooxygenase (COX), lipoxygenase (LOX), and cytochrome P450 (CYP450) and the endocannabinoids pathway [13]. Non enzymatic pathway is the oxidation of free AA in the ROS-rich microenvironment [14] (Fig. 3). AA metabolites are involved in a wide range of physiological processes, including inflammation, immune response regulation, and tissue remodeling [15]. Elevated levels of AA and its metabolites characterize the acute phase of SCI, influencing inflammatory regulation and initiating repair mechanisms[16]. While the initial inflammatory response mediated by AA derivatives is essential for debris clearance and tissue repair, sustained inflammation paradoxically promotes chronic tissue damage, exacerbating neural degeneration and compromising functional recovery [17].

The interplay between AA metabolism and neuroinflammation has garnered significant interest for its potential role in SCI pathogenesis. Evidence indicates that dysregulation of AA-derived bioactive mediators exacerbates the inflammatory cascade and amplifies secondary injury mechanisms post-SCI. In this review, we comprehensively evaluate the mechanisms that regulate AA metabolism in SCI and the AA-derived bioactive mediators which contribute to the injury. Furthermore, we evaluate potential therapeutic strategies aimed at modulating AA metabolism to mitigate its detrimental effects and improve recovery outcomes.

## Literature search strategy and methodology

This review critically examines the role of AA metabolism in SCI, focusing on its contributions to inflammation, oxidative stress, neurodegeneration, and potential therapeutic interventions. To synthesize current understanding of AA metabolic mechanisms in SCI, we conducted a comprehensive systematic literature search using PubMed and Web of Science with the following search terms: (spinal cord injury) AND ((arachidonic acid) OR (cyclooxygenase) OR (lipoxygenase) OR (cytochrome P450) OR (PLA2) OR (COX-2) OR (PGI2) OR (PGD2) OR (PGJ2) OR (PGE2) OR (TXA2) OR (TXB2) OR (5-LOX) OR (12-LOX) OR (15-LOX) OR (LTA4) OR (LXA4) OR (LTB4) OR (HPETE) OR (HETE) OR (EETs) OR (anandamide) OR (2-AG)). The publication year is restricted to the range from 1980 to 2026, with no restrictions on document type or language. The literature search was conducted by Authors X.L and W.C, following a two-stage screening protocol: first, duplicate and independent screening of titles and abstracts was carried out to preliminarily exclude irrelevant studies, and any discrepancies emerging during this screening step were resolved through in-depth discussion with a third author Y.X to minimize subjective bias and ensure screening objectivity. Explicit inclusion criteria were closely aligned with the core focus of this review, specifically encompassing studies related to AA metabolic pathways, AA-associated metabolites, SCI-related inflammatory responses, SCI-induced oxidative stress, and potential therapeutic strategies targeting AA metabolism. Meanwhile, strict exclusion criteria were applied to filter out non-eligible studies, including those unrelated to AA metabolism or SCI pathology, conference abstracts, letters, or editorials that lack original experimental or analytical data, non-English literature for which no English translation is available, and studies whose full texts remain inaccessible despite reasonable efforts to retrieve them.

## cPLA2-mediated arachidonic acid release following spinal cord injury

The PLA2 enzymes catalyze the hydrolysis of the *sn*-2 ester bond in glycerophospholipids, releasing free fatty acids and lysophospholipids. The PLA2 superfamily includes several hydrolytic enzymes such as Secreted PLA2 (sPLA2), Calcium-independent PLA2 (iPLA2), Lysosomal PLA2 (LPLA2), Adipose-specific PLA2 (AdPLA2), and cPLA2. Among these isoforms, cPLA2 is the principal enzyme responsible for generating lysophospholipids and releasing arachidonic acid from membrane phospholipids following SCI. While cPLA2 plays the predominant role, other PLA2 isoforms, including sPLA2, iPLA2, and LPLA2, are also involved in the injury response, though their contributions are secondary [18].

Under physiological conditions, cPLA_2_ enzymes are kept largely silent by several homeostatic regulators such as Annexin A1 and Camodulin that limit their membrane binding or catalytic activity. cPLA2 is primarily expressed in neurons and oligodendrocytes, and its expression is significantly upregulated after SCI. After SCI, cPLA2 activation is triggered by a convergence of injury-related signals. Mechanical trauma damages neuronal and glial membranes, causing the release of excitatory neurotransmitters such as glutamate and NMDA; these activate ionotropic receptors, lead to Ca^2+^ influx and ROS production, and markedly elevate PLA2 activity and arachidonic acid release in cultured neurons [19]. Inflammatory cytokines (e.g., TNF-α, IL-1β), free radicals, and excitatory amino acids, induce PLA2 expression and activity after SCI [20]. NMDA receptor stimulation triggers phosphorylation of cPLA2 via NADPH-oxidase-dependent ROS signaling [21], whereas downstream MAPK pathways (ERK1/2, p38, JNK) and upstream RhoA/Rho-kinase signaling further promote cPLA2 activation [19]. Lipid mediators such as ceramide-1-phosphate or elevated Ca^2+^ also directly activate cPLA2 [20]. Consequently, cPLA2 becomes a nexus where excitotoxicity, inflammation, and oxidative stress converge, amplifying secondary damage through the production of arachidonic acid metabolites and further membrane breakdown.

Elevated cPLA2 activity contributes to neuronal death and oligodendrocyte demyelination, thereby exacerbating the progression of injury [22,23]. Inhibition of cPLA2, either pharmacologically with arachidonyl trifluoromethyl ketone (AACOCF3) or through genetic deletion in mice, significantly reduces tissue damage and improves behavioral recovery following SCI [22]. AACOCF3 also mitigates dendritic length loss in motoneurons after SCI, helping preserve motor neuron structure [24]. Recent studies showed that the cPLA2 activity in infiltrating monocyte-derived macrophages does not affect recovery after SCI [25], highlighting cell–type–specific effects of cPLA2. Another cPLA2 inhibitor, almityl trifluoromethyl ketone (PACOCF3), reduces ROS and NO production, thereby alleviating oxidative stress in bone marrow-derived macrophages. PACOCF3 also inhibits neurotoxic effects caused by M1 pro-inflammatory stimulation, preventing increases in lysosomal membrane permeability (LMP) and protecting lysosomal function [26]. In addition to pharmacological inhibitors, endogenous proteins also regulate cPLA2 activity. Annexin A1, an endogenous neuroprotectant, reduces inflammation and tissue damage by inhibiting cPLA2 activation in adult rats following SCI [27]. Additionally, the activation of cPLA2 is profoundly affected by oxidative stress and glutamate excitotoxicity. The extract of Ginkgo biloba (EGb761) inhibits cPLA2 in spinal cord neurons. The study shows that part of the protective effect is due to overall reduced ERK1/2 phosphorylation [28]. Furthermore, certain compounds, such as DADLE, a selective opioid receptor agonist, have demonstrated therapeutic potential in SCI by inhibiting LMP induced by cPLA2 [29]. Similarly, elamipretide, a peptide drug, alleviates pyroptosis and tissue damage by inhibiting cPLA2-mediated LMP, thereby facilitating tissue repair and improving post-SCI functional recovery [30].

Once released, AA undergoes metabolism via major enzymatic pathways, producing various bioactive mediators that play significant roles in physiological responses, including inflammation, pain, and fever [31]. The pathways of COX generate PGs and TXs, the LOX pathway produces LTs and LXs, and the CYP450 pathway leads to the formation of EETs and ω-HETEs. Fatty acid amide hydrolase (FAAH) mediated pathway reversely produce anandamide from AA. These metabolites are crucial for modulating inflammation and immune responses [[32], [33], [34]].There is also gradual non-enzymatic oxidation of AA under ROS milieu and further exacerbated the ROS-rich microenvironment.

## Cyclooxygenase (COX) pathway in spinal cord injury

### COX-1 in spinal cord injury

Cyclooxygenase-1 (COX-1) is constitutively expressed in various tissues, where it regulates key homeostatic processes including platelet aggregation, gastric mucosal protection, and maintenance of renal blood flow. Notably, it has demonstrated that COX-1 expression is robustly upregulated during the acute inflammatory phase following spinal cord injury. This upregulation contributes to the pathogenesis of secondary injury and drives pathological remodeling of the CNS architecture. Specifically, COX-1 accumulates persistently in microglia/macrophages, endothelial cells, and smooth muscle cells within the lesion core and regions of secondary injury during the early post-injury period [35]. The sustained inflammatory response triggered by acute SCI underscores COX-1′s functional role in tissue remodeling and secondary injury progression, highlighting it as a potential therapeutic target for SCI management.

### Role of COX-2 in spinal cord injury

Under normal physiological conditions, the expression of COX-2 is low and is primarily localized to neurons, especially in their cell bodies and dendrites.Under pathological conditions, COX-2 expression significant increase in neurons as well as astrocytes and microglia. As a result of this upregulation, more prostaglandins and reactive ROS are produced at the injury site, causing further damage to the tissues and aggravates the injury [[36], [37], [38]].

Researchers have demonstrated detectable COX-2 mRNA expression in spinal cord neurons occurring as soon as 2 h post-injury, along with heightened COX-2 protein levels for at least 48 h [39]. Following SCI, COX2 is predominantly expressed by neurons [16], particularly in areas involved in pain processing. COX2 is a key enzyme in the production of prostaglandins, which are lipid mediators known to play a significant role in sensitizing nociceptive neurons. The activation of COX2 in neurons contributes to the development of central sensitization, a process by which the spinal cord becomes hypersensitive to pain stimuli. This mechanism is crucial for the chronic pain that often accompanies SCI, as COX2 expression in neurons enhances the excitability of pain-related pathways, further exacerbating pain perception. Several studies have demonstrated that the inhibition of COX2 in neurons can reduce pain behavior in SCI models, suggesting that COX2 in neurons is a critical target for pain management. The upregulation of COX-2 in SCI is closely linked to inflammatory responses, as its induction is driven by various pro-inflammatory factors, including TNF-α, TGF-β, EGF, PDGF, IL-1, IL-2, and FGF [40]. The prostaglandins and free radicals produced by COX-2 not only promote cellular injury but also contribute to neurotoxicity and inflammation, particularly through the actions of PGE2 [41]. Additionally, COX-2 modulates the inflammatory microenvironment by regulating the release of cytokines, further amplifying the pathological response [42,43].

One of the key pathways involves the activation of COX-2/PGE2 signaling in astrocytes by D-DT and MIF, which bind to the CD74 receptor. This interaction triggers intracellular cascades mediated by MAPKs, including ERK, JNK, and p38. Activation of the CD74 receptor by D-DT and MIF upregulates COX-2 and mPGES-1, leading to increased production of PGE2. Elevated PGE2 levels subsequently drive astrocyte-mediated inflammatory responses following SCI [44,45]. Similarly, the HMGB1/ TLR4 axis plays a role in astrocytic inflammation after SCI by modulating COX-2/PGE2 signaling. Both TLR4 and COX-2 exhibit significantly elevated protein levels in astrocytes after SCI, further amplifying the inflammatory response [46]. Additionally, traumatic SCI induces excessive ATP release from injured tissues. ATP interacts with the P2Y1 receptor, a subtype of purinoceptors, triggering intracellular Ca^2+^ release, EGFR transactivation, and activation of ERK1/2. This cascade rapidly activates the release of AA and PGE2 from astrocytes [47].

SCI not only leads to motor dysfunction but is also frequently accompanied by the emergence of the neuropathic pain [[48], [49], [50]]. Increased COX-2 activity strongly relates to the development and maintenance of neuropathic pain after spinal cord injury. Inhibiting COX-2 activity proved beneficial for alleviating neuropathic pain post-SCI. For instance, intrathecal administration of the COX-2 inhibitor NS398 has been found to effectively reduce mechanical hypersensitivity in SCI models [51].

Furthermore, COX-2 may exacerbates neuropathic pain through immune-mediated mechanisms in both the central and peripheral nervous systems. In the chronic phase following SCI, alterations in COX-2 expression are associated with the production of immunoregulatory markers, which can influence pain signal transmission and contribute to the persistence of neuropathic pain [52]. Microglia polarization is important in the pathogenesis and persistence of neuropathic pain. Specifically, M1 microglial polarization is tightly linked to pain persistence while M2 polarization is associated with pain relief and repair mechanisms [53].

In a rat model of neuropathic pain, microglia-derived TNF-α was shown to induce COX-2 expression, promoting the production of PGI2 and PGE2 in spinal cord endothelial cells. These prostaglandins acting with their receptors on spinal neurons are important for neuropathic pain. The interaction of PGE2 with the EP2 and EP4 receptors of spinal neurons affects pain sensation thus, leading to pain behaviours [54,55]. However, it remains unclear whether COX-2 directly participates in microglia-mediated mechanisms of neuropathic pain.

### Prostaglandin E2

While the precise mechanisms through which COX-2 regulates inflammation remain unclear, it is widely believed that COX-2-mediated inflammation is primarily driven by the enhanced production of prostaglandins, particularly PGE2. In the CNS, PGE2 is considered one of the major COX-2-derived metabolites, especially in the context of injury or neurological diseases [56]. Astrocytes and microglia are identified as the principal producers of PGE2 in response to SCI and other forms of CNS damage (Table 1).

**Table 1:** AA Metabolites in inflammation after spinal cord injury.

Metabolites	Main distribution	Role in inflammation after spinal cord injury	Reference
PGE2	Astrocytes, microglia	Promotes IL-1β and IL-6, decreases TNFα expression	[45]
PGD2	Microglia, macrophages	Microglial and macrophages activation	[57]
15d-PGJ2	Macrophages	Enhance neuronal survival, reduce demyelination and neutrophil infiltration	[58,59]
PGI2	Endothelial	Decreases TNFα expression	[60]
LTB4	Neuron	Mediating neutrophils and monocytes/macrophages infiltration	[16,61]
LXA4	Microglia, astrocytes	Modulate microglial activation and TNF-α release	[62]
EET	Neurons, astrocytes, blood vessels	Inhibits activation of microglia and expression of IL-1β, decreases neutrophil infiltration	[63]
AEA	DRG neuron	Regulate nociceptor activity	[64]

The failure of myelin sheath regeneration after SCI involve various factor whereby PGE2 is crucial for it. It has been shown that activation of ASIC1 inhibits neural stem cell differentiation into oligodendrocytes by upregulating PTGS2 expression and promoting PGE2 release in extracellular vesicles [65]. Excessive production of PGE2 not only exacerbates the inflammatory response but also impairs the function and repair capacity of myelin cells.

The spinal dorsal horn contains various prostaglandin E2 (PGE2) receptors (EP1–EP4), which play a crucial role in sensitizing central nociceptive pathways and contributing to pain hypersensitivity[66,67]. Several studies have highlighted the specific involvement of the EP2 receptor subtype in inflammatory pain [68].

### PGI2 and TXA2

PGI2 and TXA2 have opposing roles in platelet aggregation and vascular homeostasis under physiological conditions. PGI2 induces vasodilation and prevents aggregation of blood platelets, whereas TXA2 elicits vasoconstriction and promotes platelet aggregation.

In the context of SCI, combined treatment with TXA2 synthase inhibitors and stable PGI2 analogues has been shown to improve spinal cord blood flow and mitigate ischemia [69]. PGI2 exerts cytoprotective effects by increasing tissue perfusion and inhibiting leukocyte activation. For example, the stable PGI2 derivative, iloprost, has been demonstrated to alleviate SCI severity by preventing neutrophil activation [70]. This neutrophil activation is primarily driven by TNF-α, and PGI2, secreted by endothelial cells upon sensory neuron activation, contributes to SCI repair by inhibiting TNF-α production [60].

In contrast, TXA2 is metabolized to its inactive form TXB2. After SCI, TXB2 levels in the spinal cord remain elevated for at least 72 h [71]. Excessive TXB2 production has been linked to ischemic injury in SCI models like rabbits and cats, where its elevated levels may exacerbate secondary tissue damage [72,73]. These findings underscore the importance of understanding the interplay between PGI2 and TXA2 in SCI, highlighting the potential therapeutic value of targeting these pathways to alleviate SCI-induced ischemia and inflammation.

### 15d-PGJ2

15d-PGJ2, a terminal PGD2 metabolite, demonstrates significant anti-inflammatory properties [74,75]. Functioning as an endogenous PPAR-γ agonist, 15d-PGJ2 modulates cell proliferation, inflammation, and oxidative stress. After SCI, treatment with 15d-PGJ2 has been reported to promote neuronal survival and reduce demyelination. These protective effects are likely mediated by the inhibition of NF-κB activation, upregulation of suppressor of cytokine signaling 1, and attenuation of JAK2 activation during the early post-injury phase. Additionally, 15d-PGJ2 could decrease TNF-α and IL-1β levels and prevent neutrophil infiltration at the injury site [58].

However, it is important to note that while 15d-PGJ2 exerts anti-inflammatory benefits at lower concentrations, high levels of 15d-PGJ2 may paradoxically lead to increased cell apoptosis and exacerbate inflammatory responses [76]. At lower concentrations, 15d-PGJ2 has been shown to improve neuronal survival, enhance 5-HT innervation in the ventral horn, and reduce demyelination [59,77]. These findings highlight the dual roles of 15d-PGJ2 in SCI, suggesting that its therapeutic potential may be concentration-dependent. Further studies are required to better understand the underlying mechanisms and optimize the use of 15d-PGJ2 as a treatment for SCI.

### Inhibitors of COX in SCI

Inhibiting COX-2 has been shown to reduce inflammation, preserve spinal cord tissue, and improve outcomes [78,79]. For example, celecoxib reduces the concentrations of pro-inflammatory prostaglandins like PGE2 and TXB2 in the injured spinal cord [80]. Early administration of celecoxib within 5 days after TBI is associated with a higher 1-year survival probability and fewer complications [81].

The novel COX-2 inhibitors such as compound 7 m also reduce oxidative stress, alleviation of neuronal edema and necrosis [82]. Similarly, SC58125, another selective COX-2 inhibitor, has shown efficacy in improving functional recovery after SCI [83]. Proposed mechanisms include cyclooxygenase activity generating hydroxyl radicals, leading to lipid peroxidation, thus intensifying spinal cord damage. Additionally, prostaglandins produced by COX-2 may contribute to SCI pathology through vasoconstriction, platelet aggregation, and chemotaxis, as well as inducing apoptosis through thromboxane or EP3 receptor activation.

Non-selective cyclooxygenase inhibitors have been effective in reducing secondary injury in animal SCI models. However, the clinical potential is limited due to various adverse events that they produce, such as inhibited platelet aggregation and gastrointestinal atrophy by COX-1 inhibition. In contrast, selective COX-2 inhibitors do not exhibit these toxicities, as COX-1 is predominantly expressed in platelets and gastrointestinal tissues. Therefore, highly selective COX-2 antagonists may represent a safer and more effective option for SCI patients. Further research is necessary to confirm the neuroprotective mechanisms of COX-2 inhibitors and optimize their therapeutic potential in SCI.

Produced by hematopoietic prostaglandin D synthase (HPGDS), PGD2 has been identified as detrimental in SCI. Inhibition of HPGDS using HQL-79 reduces microglial activation and promotes motor function recovery [57]. Additionally, Iloprost, a stable PGI2 analog, mitigates motor disturbances and intramedullary hemorrhages in rats with spinal cord trauma by reducing leukocyte accumulation at the injury site [70].

Antithrombin reduces the increase of TNF-α, IL-8, and MPO by promoting the release of PGI2 from endothelial cells after SCI [84]. Chronic SCI stages are characterized by ongoing inflammation, with elevated levels of lipid mediators like LTB4 and PGE2 at the injury site. Using the COX/5-LOX dual inhibitor licofelone enhanced the level of endogenous antioxidant and anti-inflammatory metabolites [85]. Licofelone alleviates neuroinflammation and improves mitochondrial homeostasis by enhancing parkin-dependent mitochondrial autophagy [86].

### Lipoxygenase (LOX) pathway in spinal cord injury

LOX enzymes oxidize AA to HPETEs, which can be further converted into other bioactive lipids, such as HETEs, LTs, and LXs. The human genome contains a total of six functional LOX genes which encode different LOX isoenzymes. The three major LOX isoforms 5-LOX, 12-LOX and 15-LOX have distinct functions in AA metabolism and its derivatives.

### 5-LOX

Previous studies have shown that 5-LOX expression is elevated in neurons during the acute phase of SCI, underscoring the activation of the LOX pathway as a key factor in spinal cord injury [16]. In vascular clip compression-induced SCI models, 5-LOX-deficient mice exhibited significant neuroprotective effects [87]. The FLAP expressed by microglia plays a role in 5-LOX regulation. FLAP anchors 5-LOX to cellular membranes and facilitates its interaction with AA substrates [88]. The 5-LOX enzymatic activity is dynamically regulated through spatial reorganization and protein interactions. The lipid peroxidation scavenger edaravone can inhibit ferroptosis after SCI, an effect linked to the reduction of 5-LOX activity [89]. Elevated 5-LOX expression has been linked to ferroptosis following various CNS injuries [90,91].

### 12-LOX

Following SCI, 12-LOX expression is significantly upregulated, particularly during apoptotic cascades. 12-LOX catalyzes the conversion of AA into pro-apoptotic mediators such as 12-HETE that activate apoptotic mediators through NF-κB and p38 MAPK. These pathways drive the generation of inflammatory cytokines and upregulate Caspase-3 and Caspase-9, amplifying cellular apoptosis [92,93]. In SCI, 12-LOX-deficient models demonstrate improved behavioral recovery, further supporting the pathogenic role of 12-LOX in neuroinflammation and injury progression[92].

### 15-LOX

In the context of SCI, 15-LOX contributes to ferroptosis via the P53 signaling pathway. Activation of P53 induces the expression of 15-LOX, which through lipid peroxidation, causes aggravation of ferroptosis [94]. Researchers found that vitamin E, which inhibits 15-LOX expression, decreases ferroptosis and promotes neural function recovery following SCI in rats [95]. Additionally, inhibiting 15-LOX activity has been shown to protect endothelial cells and maintain the BBB integrity following SCI [96].

### Leukotriene B4

5-LOX mediated LTB4 exerts its effects through two main receptors: the high-affinity receptor, BLT1, and the low-affinity receptor, BLT2. BLT1 is predominantly expressed on neutrophils, monocytes, and macrophages [97,98], while BLT2 is more widely distributed. When BLT1 is activated, it usually increases the intracellular calcium levels and chemotaxis, which promotes the migration and accumulation of leukocytes [99]. In SCI, LTB4 mediates leukocyte infiltration peaking approximately 12 h post-injury. Studies have shown that BLT1 knockout mice have lesser infiltration of neutrophils and monocyte/macrophages indicating that LTB4 is critical for early post-traumatic inflammation. Inhibition of LTB4 activity has been associated with diminished inflammatory responses in SCI [61].

### Lipoxin A4

LXA4 and LXB4, are known for their potent anti-inflammatory and pro-resolution properties in various disease models, including SCI, TBI, ischemia/reperfusion injury, ischemic stroke, depression, and cerebral malaria [[100], [101], [102], [103], [104], [105], [106]]. LXA4 has been shown to improve neurological function, reduce apoptosis, lower MDA levels, and enhance SOD activity in rabbit models of ischemia/reperfusion-induced SCI [107]. Following SCI, LXA4 interacts primarily with the G-protein-coupled receptor ALX/FPR2. LXA4 effectively modulates microglial activation and inhibits TNF-α release through ALX/FPR2, thereby reducing neuropathic pain in rodent models of spinal hemisection [62]. Interestingly, the synthesis of LXA4 is delayed after SCI, 12-HETE and 15-HETE levels were enhanced only after 14 days of injury [108]. Following SCI, LXA4 has been shown to preserve Akt phosphorylation, which is essential for maintaining the expression of Nrf2 and HO-1. Inhibition of Akt in LXA4-treated rats significantly reduces Nrf2 and HO-1 expression, suggesting that Akt is an upstream signal for LXA4-induced Nrf2/HO-1 activation [101,109]. LXA4 also inhibits oxygen–glucose deprivation/reperfusion-induced astrocyte injury through Nrf2 pathway activation to attenuate oxidative stress [110].

### Modulation of LOX pathway in SCI

Both the 5-LOX inhibitor zileuton and the Cys-LT receptor antagonist Montelukast effectively reduced TNF-α, COX2 expression and neutrophil infiltration after SCI [111,112]. Furthermore, zileuton treatment has inhibitory effects on ferroptosis and antioxidant effects in neurons [112]. Combination therapies, such as pseudohypericin (an anti-lipoxygenase agent) with zafirlukast (a Cys-LT receptor antagonist), further reduce tissue damage and neutrophil infiltration while lowering levels of PGE2, LTB4, pERK1/2, TNF-α, and COX-2, resulting in decreased apoptosis and improved motor function recovery [113]. Vitamin E improves motor function recovery in rats with SCI by inhibiting Alox15 expression, thus protecting neurons from ferroptosis-induced damage [95]. Targeting LTB4 signaling has therapeutic potential. Mice that receive the LTB4 receptor antagonist ONO-4057 show decreased neutrophil and monocyte/macrophage infiltration [61]. LXA4 is a specialized pro-resolving agent that blocks monocyte recruitment and cytokine secretion, resolving hyperalgesia and allodynia. Intrathecal administration of LXA4 in rat SCI models improves functional recovery and suppresses apoptosis signaling [101], underscoring its neuroprotective potential. BML-111, an LXA4 receptor agonist, has been demonstrated to modulate inflammation and oxidative stress and improve recovery from SCI. Treatment with BML-111 decreased TNF-α, IL-1β, and IL-6 levels in serum and spinal cord tissue, while also reducing cell apoptosis [114].

### Cytochrome P450 (CYP450) pathway in spinal cord injury

Soluble epoxide hydrolase (sEH) is an endogenous key enzyme in the metabolic conversion and degradation of P450 eicosanoids called epoxyeicosatrienoic acids (EETs). Inhibition of sEH with AUDA enhances angiogenesis, reduces reactive astrocyte proliferation, microglial activation, and IL-1β expression, mitigates neutrophil infiltration, and reduces neuronal apoptosis, ultimately leading to improved motor function recovery after SCI [63].

Recent study reported cytochrome P450 family 1 subfamily B member 1 (cYP1B1). additionally, network pharmacology and molecular docking analyses confirmed CYP1B1 as a viable therapeutic target for SCI [115]. Based on analysis of single–cell RNA sequencing datasets demonstrated an increase in cYP1B1 expression within spinal cord fibroblasts following SCI. Furthermore, quercetin (Que) was shown to inhibit cYP1B1 expression and reduce nP (based on mechanical paw withdrawal threshold and thermal paw withdrawal latency) in SCI (Fig. 2).

**Fig. 1:**
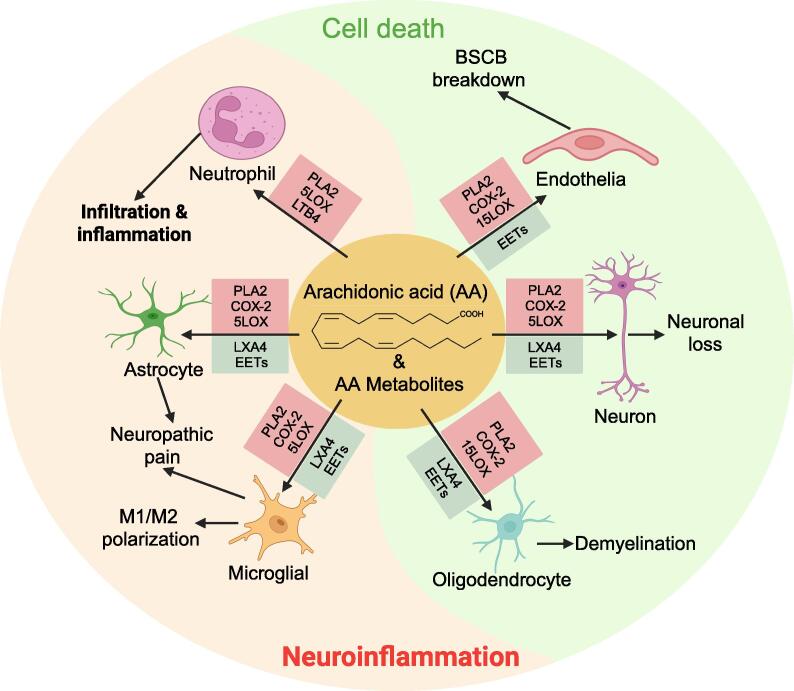
The mechanism diagram of arachidonic acid causing cell death and neuroinflammation after spinal cord injury.

**Fig. 2:**
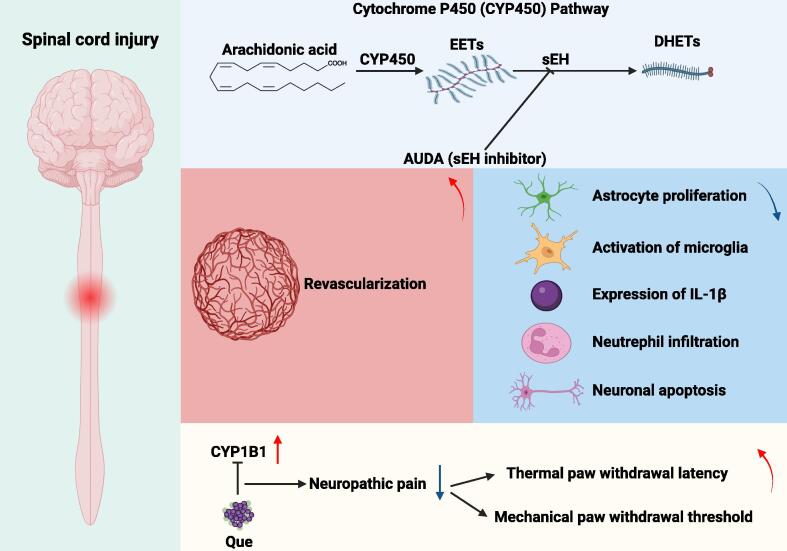
The Cytochrome P450 pathway of arachidonic acid in SCI.

**Fig. 3:**
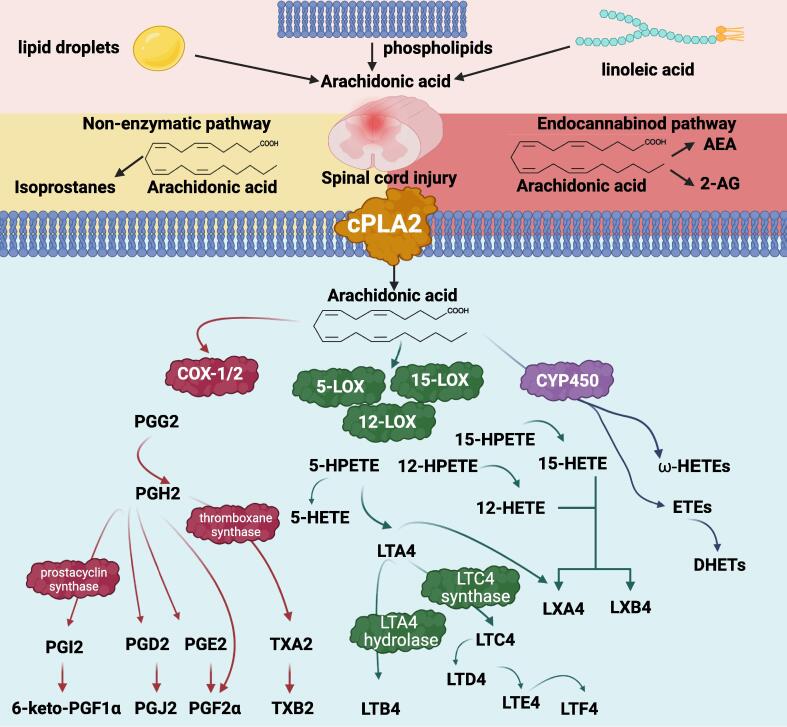
Overview of arachidonic acid in SCI.

### Endocannabinoid pathway

**Fig. 4:**
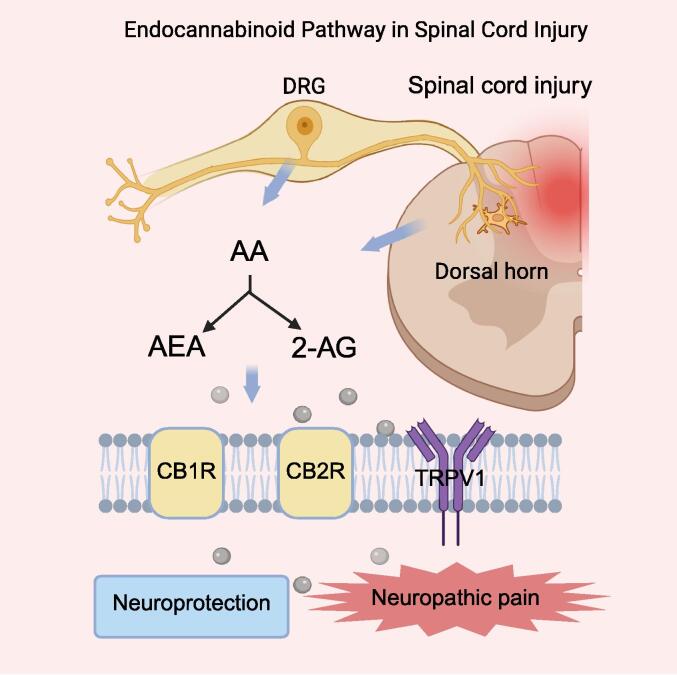
Endocannabinoid pathway in spinal cord injury. Elevated arachidonic acid following spinal cord injury can be metabolized into anandamide (AEA) and 2-arachidonoylglycerol (2-AG). These endocannabinoids exert neuroprotective effects while also inducing neuropathic pain, via cannabinoid type 1 receptor (CB1R), cannabinoid type 2 (CB2R) receptors, and transient receptor potential vanilloid 1 (TRPV1) receptor expressed on neurons in the spinal dorsal horn and dorsal root ganglia (DRG).

The endocannabinoid pathway is the fourth enzymatic pathway of arachidonic acid. As a key endocannabinoid, anandamide (AEA) is reversibly synthesized from AA via FAAH [116], with its production triggered under conditions of tissue damage characterized by elevated free AA levels [117]. SCI leads to a significant increase in AEA levels in the spinal cord and dorsal root ganglia (DRG). This upregulation, together with transient receptor potential vanilloid 1 (TRPV1), contributes to the development of chronic neuropathic pain [64]. 2-arachidonoylglycerol (2-AG) is another major endocannabinoid. It has neuroprotective effects. Early administration of 2-AG after SCI protects myelin sheaths and reduces oligodendrocyte loss. This is achieved by activating cannabinoid type 1 (CB1) and type 2 (CB2) receptors [118,119] (Fig. 4). For instance, combined treatment with the AEA hydrolysis inhibitor PF04457845 and the substrate-selective COX-2 inhibitor LM4131 alleviates neuropathic pain in mice with chronic constriction injury (CCI), while also attenuating inflammatory cell accumulation in the sciatic nerve and spinal cord [120]. Findings from this CCI model provide valuable insights for SCI research.

### Non-enzymatic AA pathways in SCI

When SCI triggers significant oxidative stress, reactive oxygen/nitrogen species generation, blood-spinal cord barrier disruption, lipid release, and arachidonic acid release, non-enzymatic spontaneous oxidation occurs in addition to the enzymatic pathways (COX/LOX/CYP450), resulting in the generation of isoprostanes. Plasma and urinary levels of 8-*iso*-PGF_2_α and 15-keto-dihydro-PGF_2_α were analysed at baseline and during the ischemia–reperfusion period in experimental spinal cord ischemia [121]. Current clinical studies indicate that urinary 8-iso-PGF_2_α levels are elevated in SCI patients, suggesting that it may serve as a biomarker for oxidative damage.

## Arachidonic acid role in cell death and euroinflammation in SCI

### AA in cell death in SCI

There are numerous studies on drugs regulating AA metabolites in the neuronal apoptosis after SCI [113,122]. The Edaravone ameliated ferrotposis in SCI and reduced the 5-LOX expression in neurons [89]. In the context of SCI, 15-LOX contributes to ferroptosis via the P53 signaling pathway. Activation of P53 induces the expression of 15-LOX, which through lipid peroxidation, causes aggravation of ferroptosis [94]. Researchers found that vitamin E, which inhibits 15-LOX expression, decreases ferroptosis and promotes functional recovery following SCI in rats [95]. Additionally, inhibiting 15-LOX activity has been shown to protect endothelial cells and maintain the BBB integrity following SCI [96]. In addition, LXA4 activates the Akt/Nrf2/HO-1 signaling pathway to promote neuronal survival in primary spinal cord neurons in Erastin-induced ferroptosis [123].

cPLA2 is the principal enzyme responsible for generating lysophospholipids and releasing AA from membrane phospholipids after SCI. These lysophospholipids destabilize the lysosomal membrane, increasing LMP. Consequent lysosomal enzyme leakage into the cytosol impairs lysosomal function and autophagic degradation, leading to autophagosome accumulation and neuronal death. cPLA2 activation preferentially occurs in the lysosomal fraction within 2 h after SCI, producing lysophosphatidylcholine (LPC) and driving lysosomal dysfunction and inflammation. cPLA2 inhibition reduces lysosomal damage, restores autophagic flux, and alleviates neuronal injury [124]. Moreover, the neuroprotective effects of DADLE are mediated by its ability to inhibit cPLA2 phosphorylation, thus reducing LMP and promoting the recovery of autophagy [29]. Elamipretide, by regulating cPLA2 phosphorylation through the MAPK-P38 signaling pathway, inhibits necroptosis, enhances autophagy, and attenuates LMP in SCI [30].

### AA in neuroinflammation in SCI

AA metabolism plays a pivotal role in mediating neuroinflammation following SCI. Neurons act as both sources and targets of AA-driven inflammation. Mechanical trauma and glutamate-induced excitotoxicity trigger cPLA2 phosphorylation via ROS and MAPK signaling, promoting AA release from membrane phospholipids [22]. Early neuronal COX-2 upregulation generates PGE2 to sensitize nociceptive neurons [51]. 5-LOX elevation links to ferroptosis and inflammatory amplification [89]. Pro-resolving LXA4 protects neurons via the Akt/Nrf2/HO-1 pathway [123]. Oligodendrocyte dysfunction and demyelination are closely tied to AA metabolism. Excessive PGE2 inhibits oligodendrocyte differentiation via ASIC1/PTGS2 signaling [65]. 15-LOX-mediated lipid peroxidation induces ferroptosis. cPLA2 activation exacerbates demyelination. In contrast, cPLA2 inhibition or vitamin E-induced 15-LOX suppression preserves myelin and mitigates damage [[94], [95]]. Microglia are central mediators of AA-related neuroinflammation. They express FLAP to anchor 5-LOX, facilitating LTB4 production for leukocyte chemotaxis [88]. 5-LOX/LTB4 signaling promotes microglial M1 polarization. Microglia-derived TNF-α induces endothelial COX-2, amplifying pain-related PGE2/PGI2 release [53]. LXA4 binds to microglial ALX/FPR2 receptors to inhibit TNF-α secretion [62]. Treg-derived CTLA-4 upregulates Abcg1, reducing microglial lipid droplet accumulation and inflammatory activation [125]. Astrocytes amplify inflammation through AA pathways. ATP-P2Y1 receptor activation triggers EGFR transactivation and ERK1/2 signaling, upregulating COX-2 and mPGES-1 to enhance PGE2 production [47]. The D-DT/MIF-CD74 axis further activates astrocytic COX-2/PGE2 signaling via MAPKs, exacerbating cytokine release [44]. Astrocytic PGE2 disrupts BBB integrity. COX-2 or sEH inhibition reduces reactive astrocyte proliferation [63]. Neutrophil infiltration is a key early event in SCI inflammation. It is driven by 5-LOX-derived LTB4 binding to BLT1 receptors, inducing calcium influx and chemotaxis [99]. BLT1 knockout or LTB4 receptor antagonism reduces neutrophil accumulation [61]. Endothelial cells modulate inflammation and vascular homeostasis via AA metabolism. PGI2 secretion improves tissue perfusion by inhibiting TNF-α production and neutrophil activation [84]. Excessive TXA2 induces vasoconstriction and ischemia. 15-LOX inhibition preserves endothelial function and BBB integrity. COX-2-induced PGE2/PGI2 production links to neuronal nociceptive signaling via EP2/EP4 receptors [53].

### Arachidonic acid in pain

After SCI, most patients experience multiple types of pain [[126], [127]]. Understanding the mechanisms behind these pain types and differentiating between them is essential for selecting the most effective treatment approach. The SCI pain taxonomy was developed to address both injury-related and non-injury-related pain [128]. It includes (A) nociceptive pain, (B) neuropathic pain (at-level neuropathic pain, below-level neuropathic pain, or other neuropathic pain), (C) other pain, and (D) unknown pain.

COX-derived metabolites, such as PGE2, are well-established mediators of pain and inflammation following SCI. Increased COX-2 activity after SCI has been closely linked to the development and persistence of neuropathic pain, with studies showing that selective COX-2 inhibition can reduce pain sensitivity and promote functional recovery in SCI models. Similarly, LOX products, such as LTB4, significantly contribute to neuroinflammation and pain persistence. Activation of the LOX pathway exacerbates immune cell infiltration and enhances pain behaviors in neuropathic SCI models. Furthermore, CYP450-derived metabolites, including EETs, have shown protective anti-inflammatory effects, suggesting a potential therapeutic avenue for modulating pain and inflammation in SCI. These findings highlight that targeting multiple AA metabolic pathways—COX, LOX, and CYP450—may offer promising strategies for improving SCI pain management and recovery outcomes.

In summary, the arachidonic acid metabolism and pathway involve in various cell types such as neuron, oligodendrocyte, microgliosis, astrogliosis, and pathology process in SCI, such as neuronal loss, demyelination, BSCB breakdown, and leads to clinical outcomes such as loss of motor and sensory function below the lesion level, neuropathic pain (Fig. 1).

## Clinical implications of AA pathway in SCI

### Summary of compound in preclinical studies of AA pathway in SCI

There is involvement of cPLA2, prostaglandins, leukotrienes, thromboxanes, and other AA derivatives in the pathophysiology of SCI. Targeting these metabolites presents a promising therapeutic strategy for SCI (Table 2). Although preclinical studies provided promising results, translating these findings into practical human therapies will require well-executed clinical trials. Currently there is no clinical trials on these drugs. If clinical trials is initiated, not only motor function but also pain should be carefully evaluated.

**Table 2:** Drugs associated with AA metabolism in spinal cord injury.

Compounds	Targets	Species/Gender	Experimental Model	Administration Method	Administration Dosage	Outcome	Reference
AACOCF3	cPLA2	C57BL/6 mice Male	Contusion	Intravenous and intraperitoneal injection	50 µL (4 mM) intravenously at 30 min and 200 µL (4 mM) intraperitoneally for 2 weeks	↓cPLA2 activity ↓PGE2 ↑Na^+^-K^+^-ATPase activity ↓MPO activity	[22]
AACOCF3	cPLA2	C57BL/6 mice Male	Contusion	Intravenous and Intraperitoneal injection	50 µL (4 mM) intravenously at 30 min and 200 µL(4 mM) intraperitoneally for 2 weeks	↓Motoneuron atrophy ↑Dendritic length ↑Vastus lateralis muscle weights	[24]
Annexin A1	cPLA2	SD rats Female	Contusion	Intraspinal injection	5 or 20 ug/rat around the injury epicenter within 5 min post-SCI	↓cPLA2 activity ↓MPO activity ↓IL-1β, Caspase-3, GFAP ↑Axon sparing ↑tcMMEP Responses	[27]
DADLE	cPLA2	C57BL/6 mice Female	Contusion	intraperitoneal injection	16 mg/kg/day, for 3 days	↑autophagic flux ↓LMP ↓necroptosis ↑AMPK/SIRT1/p38	[29]
Elamipretide	cPLA2	C57BL/6 mice Female	Contusion	intraperitoneal injection	5 mg/kg/day, for 3 days	↑autophagic flux ↓LMP ↓pyroptosis ↓p38-MAPK	[30]
Celebocid	COX-2	LE rats Male	Contusion	Orogastric tube	3 mg/kg within 20 min of injury	↓PGE2, TXB2	[80]
Celecoxib derivatives	COX-2	SD rats Male	Contusion	Intraperitoneal injection	Once daily for 14 days at doses of 2.5, 5, or 10 mg/kg.	↓COX-2, iNOS, Bax, TNF-α, IL-1β, IL-6, MDA ↑Bcl2, GSH, SOD	[82]
SC58125	COX-2	LE Rats Male	Contusion	Orogastric tube	3 mg/kg 15 min after trauma	↓COX-2	[83]
HQL-79	PGD2	C57BL/6 mice Female	Contusion	Subcutaneous injections	50 mg/kg body weight	↓PGD2, CXCL10, Mac-2 ↑IL-6, TGFβ-1	[57]
Iloprost	PGI2	Wistar rats Male	Compression	Femoral vein injection	100 ng/kg per minute continued for 3 h	↓Neutrophil infiltration	[70]
Antithrombin	PGI2	Wistar rats Male	Ischemia/reperfusion	Intravenous	250 U /kg 30 min prior to ischemia	↓Neutrophil infiltration ↓TNF-α, IL8, MPO	[84]
Licofelone	COX/5-LOX	SD rats Female	Contusion	Oral gavage	50 mg/kg Licofelone at 3 h and 18 h	↓PGE2 ↓Mechanical hypersensitivity	[85]
Zileuton	5-LOX	CD1 mice Male	Compression	Intraperitoneal injection	50 mg/kg at 1 h and 6 h after injury	↓Neutrophil infiltration ↓TNF-α, Bax, COX-2, pERK1/2, PGE2, LTB4 ↑Bcl-2	[111]
Montelukast	Cys-LT receptor	CD1 mice Male	Compression	Intraperitoneal injection	5 mg/kg at 1 h and 6 h after injury	↓Neutrophil infiltration ↓Apoptosis ↓TNF-α, Bax, COX-2, pERK1/2, PGE2, LTB4 ↑Bcl-2	[111]
Pseudohypericin and zafirlukast	5-LOX Cys-LT receptor	CD1 mice Male	Compression	Oral administration	PHP (120 μg/kg), ZFL (10 mg/kg) twice a day	↓Neutrophil infiltration ↓Apoptosis ↓TNF-α, FAS-L, Bax, COX-2, pERK1/2, PGE2, LTB4 ↑Bcl-2	[113]
Vitamin E	15-LOX	SD rats Female	Contusion	Intraperitoneal injection	100 mg/kg for 3 consecutive days after the operation	↓Iron overload ↓15-lox, Ptgs2 and 4Hne	[95]
ONO-4057	LTB4 receptor	C57BL/6J mice Female	Contusion	Intraperitoneal injection	10 mg/kg every 24 h for the first 5 days after injury	↓Neutrophil and monocyte/macrophage infiltration ↓IL-6, IL-1β, TNFα, CXCL1, CXCL2, CCL2, Caspase 8	[61]
LXA4	Akt/Nrf2/HO-1 signaling pathway	SD rats Male	Contusion	Intrathecal injection	300 pmol at 4 h and 24 h after injury	↑P-Akt, Nrf2, HO-1	[101]
BML-111	LXA4 receptor	SD rats Male	Compression	Intraperitoneal injection	1 mg/kg	↓TNF-α, IL-1β, IL-6 ↓TOS, TAS ↓apoptosis	[114]
AUDA	Soluble epoxide hydrolase	SD rats Male	Contusion	Intrathecal reagent infusion	1 mM for 7 days	↓Reactive astrogliosis ↓Activation of microglia, IL-1β ↓Neutrophil infiltration ↓Neuronal death ↑Angiogenesis, GAP-43	[63]

### Dieatary ω-3 and ω-6 PUFA

ω-3 PUFAs, such as eicosapentaenoic acid (EPA) and docosahexaenoic acid (DHA), have anti-inflammatory properties and can modify the activity of the AA pathway. ω-3 PUFAs compete with ω-6 PUFAs for incorporation into cell membranes, leading to the production of less inflammatory eicosanoids [129]. Additionally, ω-3 PUFAs can modulate the expression of pro-inflammatory cytokines and reduce oxidative stress, both of which contribute to the resolution of inflammation and promotion of tissue repair after SCI [130]. These ω-3 PUFA also use the COX LOX system to produce anti-inflammatory mediators. The blockade of COX LOX may also interfere with the beneficial effect of ω-3 PUFAs.

### NSIAD in pain with SCI

NSAIDS has many side effects such as irritation of gastrointestinal (GI) tract ulcers, bleeding, and gastritis[131]. In SCI patients, especially those with reduced mobility and the potential for pre-existing GI issues, the risk could not be ignored. NSAIDs are known to potentially impair renal function by reducing renal blood flow, which can be problematic in SCI patients who may already have compromised kidney function due to trauma or dehydration. Prolonged use of NSAIDs can increase the risk of cardiovascular events, such as heart attacks and strokes[132]. SCI patients, particularly those with comorbid conditions such as hypertension or metabolic syndrome, may face heightened cardiovascular risks.

### NSAID prophylaxis for heterotopic ossification in spinal cord injury

Heterotopic ossification (HO) with clinical significance develops in approximately 20–30 % of patients with spinal cord injury [133], substantially impeding their rehabilitation process. The efficacy of NSAIDs for preventing HO following total hip arthroplasty has been well-established in the literature [134]. Two randomized controlled trials have demonstrated the effectiveness of indomethacin and rofecoxib as primary prophylactic agents against HO in patients with SCI [135,136]. Additionally, a retrospective study suggested that NSAID prophylaxis may facilitate the prevention of HO development during the acute phase of SCI [137]. However, further prospective studies are warranted to optimize the prophylactic potential of NSAIDs for this patient population.

### AA metabolites as biomarkers in spinal cord injury

Metabolomics has shown considerable promise for furthering SCI research in recent years. Using high-performance liquid chromatography-tandem mass spectrometry, our previous study has demonstrated a significant increase in AA metabolites such as PGE2 and LTB4, within spinal cord tissue, during the acute phase of SCI [16] (Table 3). However, the metabolic changes of AA during the chronic phase of SCI remain insufficiently explored and warrant further investigation.

**Table 3:** Arachidonic acid metabolomics reveals elevated metabolites levels post-SCI.

Time points	Elevated metabolites of arachidonic acid
4 h	PGD2, PGE2, LTB4, 12-HETE, TXB2
24 h	PGE2, LTB4, 9-HETE
48 h	PGE2, LTB4, 6k-PGF1a, TXB2, 5-HETE, 8-HETE, 11-HETE, 15-HETE, LXA4, 5,6 DHET, 8.9 DHET, 11,12 DHET, 14.15 DHET

PGE2 concentrations in CSF show correlation with SCI severity and recovery. Clinical and preclinical observations support this: Canine SCI studies reveal CSF PGE2 fluctuations corresponding to injury severity and recovery [138], while rat models demonstrate peak CSF PGE2 concentrations at 4 h post-injury [139]. These findings position AA metabolites, especially PGE2, as promising biomarkers for assessing SCI severity and tracking recovery.

## Future directions in arachidonic acid research for spinal cord injury

Both mechanisms as well as clinical approaches need to explore in the future direction of arachidonic in SCI. The following are mechanical study as well as clinical approaches respectively which AA may involve in the frontier of SCI innovation and translation.

### How AA pathway involves in Lipid Droplet in SCI

Abnormalities in AA metabolism following SCI are also closely associated with lipid metabolic disorders. The increase PU.1 expression causes lipid droplet accumulation as well as the formation of cholesterol crystals inside macrophages and microglia[140]. Regulating or targeting PU.1 expression may limit deposition of lipid metabolic waste at the site of injury, thus promoting SCI repair and recovery. Regulatory T (Treg) Treg cell upregulated the ATP-binding cassette transporter G1 (Abcg1) receptor in microglia, mitigated microglial inflammation via CTLA-4 and manage myelin load and reduce lipid droplet formation [125]. The role of AA metabolism in lipid droplets in SCI is less understood.

### AI-powered database for arachidonic acid metabolites as biomarkers in spinal cord injury

AA metabolites play a crucial role in SCI by influencing inflammation and tissue damage. An AI-powered database could integrate AA metabolite profiles from plasma, urine, and cerebrospinal fluid (CSF) to classify SCI severity and predict recovery outcomes [141]. Using machine learning algorithms, the database would analyze metabolite patterns to categorize SCI into severity levels (mild, moderate, severe) and track changes over time [142]. This system would support personalized treatment strategies by correlating specific metabolite levels with therapeutic options, such as COX-2 or LOX inhibitors. Longitudinal monitoring through the database would allow clinicians to assess treatment efficacy and adjust care plans [143]. However, challenges such as standardizing sample collection, ensuring data quality, and integrating clinical data must be addressed. Despite these obstacles, this AI-driven approach could significantly improve SCI diagnosis, monitoring, and treatment, offering valuable insights for clinicians to optimize patient care.

### Development of a multimodal pain biomarker platform integrating brain-computer interface and COX-2 PET-CT imaging for precision pain management

We aim to integrate Brain-Computer Interface (BCI) technology with COX-2 metabolites measured through PET-CT imaging, to develop a cutting-edge multimodal pain biomarker platform that will drive a transformative breakthrough in pain medicine. By utilizing BCI to decode real-time brain activity associated with pain and coupling this with COX-2 PET-CT imaging to precisely monitor the metabolic processes of arachidonic acid, this platform will capture the biological signatures of pain with unprecedented depth and accuracy [144]. This innovative multimodal integration will allow for the precise identification of different types and severities of pain, providing clinicians with novel, personalized pain assessment and treatment strategies [145]. The platform has the potential to address complex clinical challenges related to chronic pain, acute pain, and neuropathic pain by offering refined diagnostic tools [146]. Furthermore, it will provide a groundbreaking approach to pain mechanism research and therapeutic optimization. By merging the latest advances in neuroscience and molecular imaging, this platform will empower the precise and individualized treatment of pain, marking the beginning of a new era in pain management.

## Conclusions

The metabolites of AA are crucial in mediating the inflammatory and pain responses after SCI. Enzymes like COX and LOX process AA generate signaling molecules that contribute significantly to pathophysiological processes of SCI. These bioactive lipids influence secondary injury cascades by driving inflammation, exacerbating oxidative stress, and triggering cellular death. A deeper understanding of how AA and its metabolites propel SCI pathology is essential for identifying new therapeutic targets. Although cPLA2 inhibitors, COX inhibitors, and LOX inhibitors have demonstrated efficacy in preclinical models, their safety and effectiveness in humans require confirmation through further clinical studies. The complex interplay among these metabolites suggests that simultaneously targeting multiple pathways within the AA metabolic network might yield greater therapeutic benefits compared to focusing on a single pathway. Furthermore, the role of AA metabolites in modulating inflammation and pain underscores the promise of combinatory therapeutic strategies designed to address these interconnected pathways. In summary, AA metabolites are crucial contributors to SCI pathology. While current therapeutic approaches targeting these metabolites show promise, additional research is required to optimize these strategies, enhance their clinical translation, and ultimately improve recovery outcomes for SCI patients.

## Compliance with ethics requirements

Our article does not contain any studies with human or animal subjects.

## CRediT authorship contribution statement

**Xue Yao:** Writing – review & editing, Conceptualization. **Xinjie Liu:** Writing – original draft. **Wenbin Cai:** Writing – original draft. **Guangzhi Ning:** Writing – review & editing. **Xu Zhang:** Writing – review & editing. **Kailiang Zhou:** Writing – review & editing. **Shiqing Feng:** Writing – review & editing, Funding acquisition.

## Declaration of competing interest

The authors declare that they have no known competing financial interests or personal relationships that could have appeared to influence the work reported in this paper.
